# F250. DEPRESSION IN SCHIZOPHRENIA: CORRELATIONS WITH OBJECTIVE AND SUBJECTIVE QUALITY OF LIFE OUTCOMES

**DOI:** 10.1093/schbul/sby017.781

**Published:** 2018-04-01

**Authors:** Philip Harvey, Kimberly Vanover, Robert Davis, David Penn, Amy Pinkham

**Affiliations:** 1 Leonard M. Miller School of Medicine, University of Miami; 2 Intra-Cellular Therapies, Inc; 3 University of North Carolina; 4 University of Texas at Dallas

## Abstract

**Background:**

Schizophrenia is increasing recognized to be associated with symptoms of depression. As many as 40% of people with schizophrenia (SCZ) have a fully syndromal major depressive episode at some time in their lives and the mean severity of depression in unselected samples is often in the mildly to moderately depressed range (mean BDI score of 11–16). While patients who report no depression have been found to report very low levels of subjective distress, a comprehensive study of the subjective quality of life correlates of depression in schizophrenia has not been performed. Further, given the impact of depression on interpersonal functioning, an assessment of the relationships between depression and social cognition is warranted.

**Methods:**

Two samples of patients with SCZ (n’s= 179 and 218) were compared to samples of HC (n’s= 104 and 154) and were examined with self-reported measures of depression (Beck Depression Inventory-II; BDI), social cognition, and everyday functioning and performed a total of 14 different social cognition performance-based tests. Some of these tests measured attribution bias (AIHQ), while others measured interpersonal sensitivity (PADS, PID5) while others were performance based tests of emotion recognition and perception as well as social inference and theory of mind. Participants were also examined for their speed of completion of the tasks and their confidence in their accuracy. Patients were also clinically rated with the PANSS.

**Results:**

In both samples, SCZ patients were more depressed than HC (15,15, vs. 6 and 6). In both samples of SCZ, BDI scores were correlated with clinical ratings of depression (PANSS item 6: r’s=.60 and .61. Performance on tests of emotion recognition and perception, social inference, and theory of mind were not correlated with BDI in either sample. In both samples, higher BDI were correlated with self-reports of more impaired everyday functioning, lower subjective impressions of social cognitive competence, and greater feelings of interpersonal sensitivity, combined with the impression that others were mistreating them. Depression in HC, but not patients, was associated with lower confidence while performing social cognitive tests and depression in SCZ, but not HC, was associated with slower performance on these same tests.

**Discussion:**

Depressed mood impacts self assessment of abilities and global world views in very similar ways in HC and people with SCZ. These impressions are not due to objective impairments in performance that are associated with depression. In contrast, objective performance on social cognitive tests, like previous studies of the relationship of neurocognition and functional and depression, shows remarkably little overlap with subjective depression. Although the similarity of the relationships between depression, interpersonal sensitivity, and subjective quality of life are similar in HC and SCZ, the more severe depression on the part of the SCZ populations suggests that this is an area of considerable importance for clinical intervention with either pharmacological or psychotherapeutic interventions.

